# The Value of Intraoperative Ultrasound in Selective Lateral Cervical Neck Lymphadenectomy for Papillary Thyroid Cancer: A Prospective Pilot Study

**DOI:** 10.3390/cancers13112737

**Published:** 2021-05-31

**Authors:** Giovanna Di Meo, Francesco Paolo Prete, Giuseppe Massimiliano De Luca, Alessandro Pasculli, Lucia Ilaria Sgaramella, Francesco Minerva, Francesco Antonio Logoluso, Giovanna Calculli, Angela Gurrado, Mario Testini

**Affiliations:** 1 Unit of Academic General Surgery “V. Bonomo”, Department of Biomedical Sciences and Human Oncology, University of Bari, Polyclinic Hospital, 70124 Bari, Italy; francesco.prete@policlinico.ba.it (F.P.P.); max-de-luca@libero.it (G.M.D.L.); alessandro.pasculli@uniba.it (A.P.); ilaria.sgaramella@policlinico.ba.it (L.I.S.); giovannacalculli@hotmail.it (G.C.); angela.gurrado@uniba.it (A.G.); mario.testini@uniba.it (M.T.); 2 Unit of Academic Internal Medicine “A. Murri”, Department of Biomedical Sciences and Human Oncology, University of Bari, Polyclinic Hospital, 70124 Bari, Italy; francescominerva@hotmail.it; 3 Unit of Academic Endocrinology, Department of Emergency and Organ Transplantations, University of Bari, Polyclinic Hospital, 70124 Bari, Italy; francesco.logoluso@aslbat.it

**Keywords:** neck dissection, intra-operative ultrasound, metastatic papillary thyroid cancer

## Abstract

**Simple Summary:**

Selective neck dissection is currently accepted as the standard of care for lateral cervical nodal disease from papillary thyroid cancer. However, the clinical intraoperative detection of metastatic nodal involvement may be challenging, especially in difficult-to-spot neck levels behind the sternocleidomastoid muscle, and hinges on imaging investigations. In this respect, the significance of diagnostic intraoperative US in metastatic papillary thyroid cancer seems to be under-investigated in literature. We ran a prospective diagnostic study where an intraoperative ultrasound (IOUS) showed increased sensitivity for the detection of lateral neck nodes with respect to preoperative US scans, especially in the difficult-to-spot neck levels: in sublevels such as IIb and V, up to one quarter of patients examined had nodes newly detected by IOUS that were confirmed at pathology. This study offers evidence that IOUS may provide accurate intraoperative node mapping, supporting precise and more reliable selective neck dissection.

**Abstract:**

(1) Background: Lymph node metastases from papillary thyroid cancer (PTC) are frequent. Selective neck dissection (SND) is indicated in PTC with clinical or imaging evidence of lateral neck nodal disease. Both preoperative ultrasound (PreUS) and intraoperative palpation or visualization may underestimate actual lateral neck nodal involvement, particularly for lymph-nodes located behind the sternocleidomastoid muscle, where dissection may also potentially increase the risk of postoperative complications. The significance of diagnostic IOUS in metastatic PTC is under-investigated. (2) Methods: We designed a prospective diagnostic study to assess the diagnostic accuracy of IOUS compared to PreUS in detecting metastatic lateral neck lymph nodes from PTC during SND. (3) Results: There were 33 patients with preoperative evidence of lateral neck nodal involvement from PTC based on PreUS and fine-needle cytology. In these patients, IOUS guided the excision of additional nodal compartments that were not predicted by PreUS in nine (27.2%) cases, of which eight (24.2%) proved to harbor positive nodes at pathology. The detection of levels IIb and V increased, respectively, from 9% (PreUS) to 21% (IOUS) (*p* < 0.0001) and from 15% to 24% (*p* = 0.006). (4) Conclusions: In the context of this study, IOUS showed higher sensitivity and specificity than PreUS scans in detecting metastatic lateral cervical nodes. This study showed that IOUS may enable precise SND to achieve oncological radicality, limiting postoperative morbidity.

## 1. Introduction

Lymph node (LN) metastases from papillary thyroid cancer (PTC) may occur in up to 90% of cases [1], with metastasis in the lateral neck compartment affecting the recurrence rate of PTC [2]. Radical or modified radical neck dissection has historically been the standard of care for patients presenting with LN metastases from PTC [3]. Developed by the late 20th century, the term selective neck dissection (SND) refers to a type of neck dissection in which one or more NLs normally removed in a radical neck dissection are preserved, resecting only the nodal groups at the greatest risk for metastasis from the primary site. SND is indeed described with respect to the NLs removed according to the 2002 classification [4] and is now widely employed for elective treatment and staging [5,6]. On the other hand “node picking”, LN ‘‘plucking’’ or ‘‘berry picking’’ implies the removal only of the clinically involved LNs rather than a complete nodal group within the compartment, an obsolete practice that is not recommended [7,8]. SND depends on the reliable identification of pathologic neck lymph nodes. The most common lymph nodes to harbor metastases from PTC are levels III and IV [9], while levels II and V are usually resected if there is evidence of the disease [10]. The clinical detection of nodal involvement may be difficult for node levels (NLs) located behind the sternocleidomastoid muscle; moreover, the dissection of stations II and V is potentially affected by a significant risk of complications.

High-resolution cervical US has been reported to be the most sensitive method for detecting nodal metastatic disease as small as 2 to 3 mm in diameter in patients with PTC [11,12,13,14]. While preoperative US (PreUS) certainly contributes to the success rate of lymph node excision, it has also shown, at its best, to potentially miss up to 80% of small lymph node metastases that may manifest later as recurrence/persistent disease [15], and its ability to detect nodal disease may be impacted by the patient’s position and compliance [16]. There is evidence that intraoperative US (IOUS) may provide a more accurate and reliable node mapping, especially of the difficult-to-spot NLs and sublevels [17], aiding the surgical strategy [16]. The significance of diagnostic IOUS in metastatic PTC seems, however, to be under-investigated [16]. We conducted a prospective diagnostic study focused on the use of IOUS in the context of elective SND for PTC.

## 2. Materials and Methods

### 2.1. Study Design and Patients

A prospective diagnostic study was designed around the research question: (P) In a population of adult patients with lateral neck nodal metastasis from papillary thyroid cancer, can (I) IOUS offer a higher diagnostic accuracy compared to (C) PreUS in (O) detecting metastatic lateral neck lymph node compartments?

This study was approved by the Independent Ethical Committee of the University of Bari (Protocol n.19/5758) and was performed in accordance with the Declaration of Helsinki of 1975, revised in 2013. All patients gave their informed written consent before participating in the study. Patients who were diagnosed with PTC with preoperative evidence of involvement of the lateral neck lymph nodes (LNs), based on clinical examination or PreUS, were offered participation in the study. The diagnostic approach to nodal metastasis from thyroid cancer is protocolized among the Units of Endocrinology, Radiology, General Surgery and Pathology. A preliminary consensus among all physicians taking part in this study was obtained on the nodal levels’ classification proposed by the American Head and Neck Society and the American Academy of Otolaryngology–Head and Neck Surgery [4]. Only imaging performed by radiologists with proven experience in neck US was admitted for this study. The confirmation of lateral neck node involvement on fine-needle aspiration cytology (FNAC) was a requirement for all participants.

Exclusion criteria were a refusal of consent to the study, neck dissection for malignancy other than PTC and reintervention on the lateral neck. The physician performing IOUS was aware of this study but not informed of the report of the preUS, as they were first called into the operating room before the incision to perform the pre-incision IOUS.

This study focused on the outcomes of IOUS performed only on LC nodes. The lymphadenectomy specimens were analyzed at the Pathology Unit of our center.

The deidentified data for all the cases were entered prospectively into an IRB-approved database. The data analyzed included age, gender, BMI, operative time, pathology, size and number of LNs removed, the number and location of missed LNs detected by intraoperative US, complications and recurrences.

### 2.2. Technique

**Figure 1:**
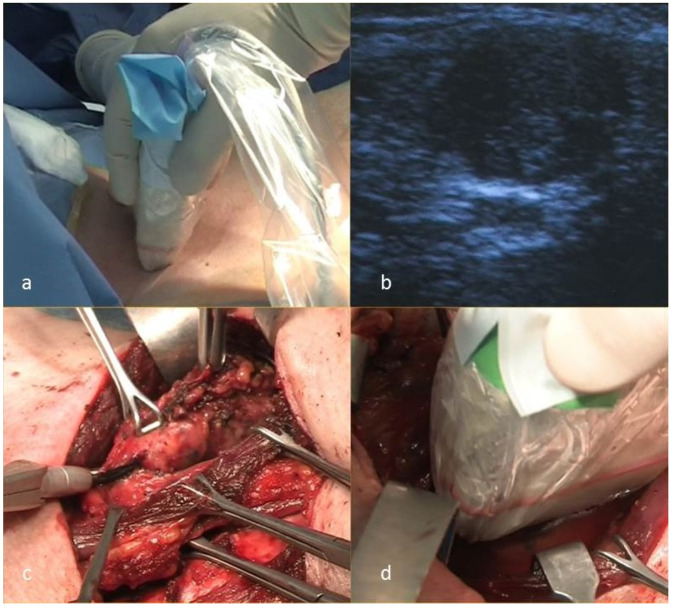
Intraoperative US and US-guided selective lateral neck dissection: (**a**) pre-incision IOUS, (**b**) US image of a lymph node with high suspicion for metastasis from PTC, (**c**) selective lateral neck dissection and (**d**) postresection IOUS.

After the induction of general anesthesia, the patient is positioned supine with the neck hyperextended, and the skin is prepped and draped. The US probe is placed in a sterile sheath, and then, pre-incisional IOUS is performed (Figure 1a). The thyroid gland is analyzed, along with the pre-tracheal, paratracheal and pre-cricoid (Delphian) nodes and the perithyroidal nodes from the hyoid bone to the suprasternal notch, including the LNs along the recurrent laryngeal nerves (level VI). With the patient’s head rotated sideways, the upper jugular level (IIa and b) is scanned, descending towards the middle and lower ones (III and IV). The probe is moved posteriorly to examine the posterior triangle (levels Va and Vb). All the abnormal NLs involved are noted (Figure 1b). SND is carried on the guidance of combined PreUS and IOUS information (Figure 1c). After lymphadenectomy, the wound is filled with sterile saline solution, and an additional IOUS is performed (Figure 1d). If any additional suspect lymphatic structure is identified at this stage, the lymphadenectomy is extended to the respective NL. Finally, postresection IOUS ensures that no sonographically detectable pathologic tissue was left. A closed suction drain is positioned.

### 2.3. Measures of Outcome

Primary outcome measures were:

- The difference, between IOUS and PreUS in the overall number of patients in which each of the lateral neck compartments containing pathologically verified metastatic lymph nodes was correctly detected.

Based on the nodal compartments correctly diagnosed, the sensitivity, specificity, positive predictive value (PPV), negative predictive value (NPV) and accuracy of PreUS and IOUS were compared.

Secondary outcome measures were:

- the overall mean lymph node yield per each compartment dissected, as a surrogate for completeness of the nodal compartment excision;
- mean operating time in minutes;
- postoperative complications rate;
- local recurrence rate during follow-up.

### 2.4. Statistical Analysis

Data were presented as the number of cases (%) or mean (range), as appropriate. For each nodal compartment, the dichotomous outcome variable “compartment positive for pathologic nodes” was examined for the independence of the observations from PreUS and IOUS, respectively, using the chi-square test (positive compartment at PreUS vs. positive compartment at IOUS). Statistical analysis was conducted using SPSS^®^ ver.22.0.0 software (IBM, Armonk, NY, USA), with the significance set at 0.05.

The diagnostic performance of PreUS and IOUS was evaluated after grouping the results of metastatic lymph node compartment detection as true positive (TP: high suspicion ± intermediate suspicion and pathology data indicating cancer), true negative (TN: very low suspicion or low suspicion ± intermediate suspicion and benign pathology data), false positive (FP: high suspicion ± intermediate suspicion and benign pathology data) and false negative (FN: very low suspicion or low suspicion ± intermediate suspicion and pathology data indicating cancer). The positive predictive value (PPV) was calculated using the formula TP/(TP + FP) and negative predictive value (NPV) with the formula TN/(TN + FN). A 95% confidence interval (95% CI) was considered in all estimates

## 3. Results

Between April 2013 and December 2019, among 445 patients who underwent neck surgery for thyroid disease and nodal metastasis from thyroid disease at our Academic Unit of General Surgery, 33 consecutive patients with lateral cervical nodal metastasis from thyroid cancer were included in this study. The demographic, surgery, pathology and follow-up data are summarized in Table 1.

All patients underwent PreUS performed by a physician with experience in endocrine pathology. Lymph nodes with suspected involvement were diagnosed on the basis of ultrasound characteristics of metastatic lymph nodes of papillary thyroid cancer, such as the presence of calcification, cystic change, loss of an echogenic fatty hilum, hyperechogenicity, round shape and abnormal vascularity on color Doppler images [18]. Lateral neck nodal compartments suspect of metastasis on PreUS were confirmed in all cases with FNA-based cytology under imaging guidance from the EcoLab Esaote and Hitachi Aloka F37^®^ ultrasound machines using 12–14-MHz transducers.

All patients underwent total thyroidectomy with central compartment lymphadenectomy. All the surgical procedures were performed by two high-volume endocrine surgeons (M.T. and A.G). Recurrent laryngeal nerve visual identification with image magnification [19] and intermittent neural monitoring were systematically carried out (NIM 3.0; Medtronic, Dublin, Ireland). Nine patients (27.3%) underwent SND after a previous total thyroidectomy. Patients underwent unilateral (*n* = 30, 91.0%) or bilateral (*n* = 3, 9.0%) SND.

**Figure 2:**
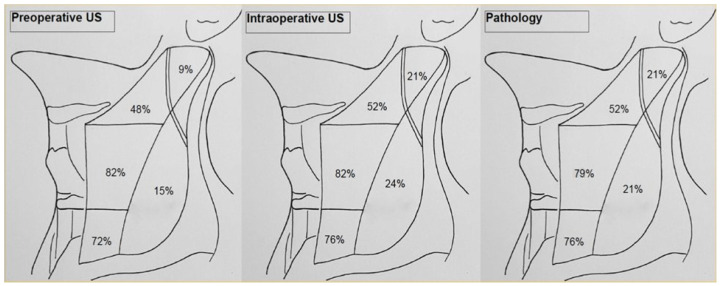
Rate of metastatic involvement of specific lateral neck nodal compartments, as found, respectively at PreUS, IOUS and the final pathology.

Table 2 and Figure 2 show positive NL recognized, respectively, at the PreUS, IOUS and final pathology. IOUS showed a higher positivity record in NL IIb and V, while no further information was added about NL IIa and IV. In particular, the levels IIb and V of PreUS vs. IOUS records rose, respectively, from 9% to 21% and 15% to 24%. Furthermore, we also found a correspondence between the IOUS findings and the final pathology and observed an increase in sensitivity from PreUS to IOUS, particularly in levels IIB and V, from 42.9% to 100% and from 71.4% to 100%, respectively. There was no perioperative mortality. The overall postoperative morbidity (i.e., recurrent laryngeal nerve palsy, definitive hypocalcemia, hemorrhage and seromas) included four patients with transient hypocalcemia (12.1%) in the post-thyroidectomy cohort. No case of shoulder syndrome or chylothorax (from injuries to the thoracic duct) was recorded.

## 4. Discussion

To the best of our knowledge, this is the first prospective study presenting data on the diagnostic performance of IOUS for lateral cervical nodal metastasis from primary PTC in the context of SND. It builds on previous evidence that surgeon-performed IOUS can help assess the completeness of modified radical neck dissection in thyroid cancer [16].

SND is accepted as a necessary therapy in patients with clinically or radiographically positive lateral compartment disease [20]. The completeness of SND contributes to the accuracy of follow-up investigations and prognostication, given that metastases in the lateral neck compartment affect the recurrence rate of PTC [2].

In the last decades, more extensive knowledge about lymphatic drainage allowed for the reliable prediction of NLs most likely to be involved with metastatic disease from each specific primary neck cancer and provided the rationale for tailored surgery such as SND [21].

To date, the ATA guidelines have assigned a significant role to suspect LN detection based on preoperative imaging investigations and intraoperative palpation or visualization, with regards to the role and extension of lateral neck dissection [2,22].

Indeed, the most often involved, and consequently explored, compartments for lateral node metastases are II and IV, although just a few studies mention in detail the sublevels of NL V and II [23,24,25].

The level V area is where the surgeon may fall short because of its relative broadness, which requires careful adherence to surgical boundaries for an adequate dissection. According to the literature reports, level V involvement ranges from 15% and 20% of neck specimens, and it is never described alone [23,26,27]. The frequency of level IIb node metastases ranges from 2.1% to 22% and are often found in association with pathological nodes in level IIa and in patients showing aggressive nodal disease [23,25,26,27]. Compartments IIb and V pose additional difficulty to the surgeon assessing the potential LN involvement at these levels, as intraoperative palpation may be difficult. This limitation may be one of the reasons why IOUS was particularly helpful in detecting pathologic LNs in levels IIa and V. Of note, all LNs indicated by a PreUS scan were confirmed by IOUS, and all compartments newly detected by IOUS but one (89%) harbored positive nodes at the pathological examination.

Overall, the IOUS indicated SND for nodal compartments that were not predicted by PreUS in nine (27.2%) cases, of which eight (24.2%) were proven to harbor positive nodes at pathology.

The IOUS can enhance the completeness of lateral neck dissections by detecting additional nodes that were missed by visual inspection or palpation, specifically in levels II, IV and V [16].

In this study there was a good correspondence between the IOUS findings and the final pathology; aside from the experience of the clinician performing the IOUS, adequate neck extension and compliance under anesthesia, and the possibility to check the operating field at the end of the dissection with the US probe, were the key factors offering an advantage to IOUS over PreUS.

Additional detections of IOUS with respect to PreUS were concentrated in NLs IIb and V, whereas no further information came from levels IIa, III and IV. The new detections at levels IIb and V helped change the operative strategy by extending the dissection to an additional NL in more than a quarter of the cases. Indeed, dissection should be extended to levels IIb and V just in case of their proven involvement, considering how less common nodal metastasis from PTC are at these levels and in view of the potential morbidity associated with dissecting these NLs. Shoulder syndrome is one major postoperative sequel of both levels IIb and V dissection, consisting of constant pain, limited active shoulder abduction and anterior flexion movements, shoulder retraction, tilt and drop and winged scapula. Injuries to the spinal accessory nerve are mainly due to blood supply interruption, traction or transection during surgery. The reported incidence of shoulder syndrome ranges from 1.5% to 27.0% after modified radical neck dissection and from 0% to 2.7% after SND [23,28,29]. Level V dissection also creates a significant dead space, significantly adding to the risk of postoperative seroma, the need for long-lasting suction drainage and residual fibrosis. Cervical sensory nerves sacrificed usually leads to widespread paresthesia and neuropathic pain, aside from increasing the postoperative stay.

These complications may be limited by increasing the reliability of SND, provided that SND is as effective as modified or radical neck dissection. In such cases, the number of lymph nodes removed in SND should also be comparable to that of the corresponding NLs in radical neck dissection [21,30]; the mean number of LNs usually found in level V dissection is usually significantly less than that of the nodes in levels II, III and IV [21]. The IOUS showed a high specificity in detecting additional pathologic nodes, and IOUS-directed compartment dissection produced a mean number of LN in the additional lateral neck compartments of 4.3, with the expected proportion of nodes found in level V; this compares well with the mean lymph node yield observed in autoptic studies [21].

These results support the reliability of IOUS, suggesting that its use may be of value in units of endocrine or general surgery where lateral neck dissection is not routinely performed, as in specialized head and neck divisions.

The evidence produced by this study needs to be framed in the context of limitations, as that of a relatively small and composite series of cases and the lack of a long-term follow-up for all cases, to weigh the true clinical advantage of a change in the operative strategy. Nevertheless, with the methodological advantage of a prospective design, it offers a detailed account of the metrics of the IOUS diagnostic power for each of the lateral neck nodal stations that were investigated and presents with a significant effect size of the IOUS in terms of the diagnostic detection of nodal stations, with a quarter of the patients correctly diagnosed due to a change in the operative strategy.

## 5. Conclusions

This study presented our initial experience with SND aided by the IOUS. In the context of this study, the IOUS operated by a clinician with experience in endocrine pathology showed a significantly higher sensitivity and specificity than the preoperative US scan alone in detecting the pathological nodal compartments, specifically in levels IIb and V, where, otherwise, systematic dissection can impact on the morbidity. The IOUS helped guide the neck dissections, changing the operative strategy in one out of four patients. This study offered evidence that the IOUS may provide accurate intraoperative node mapping, supporting precise SND, and may be of particular value to the units of general or endocrine surgery where radical lateral neck dissection is not routinely performed.

## Figures and Tables

**Table 1:** Demographics and clinicopathological data.

Demographics and Data		N (%)
Total cases		33 (100)
Gender		
F		24 (73)
Mean BMI (range)		26.6 (17–36)
Mean age (range), years		46 (20–77)
	<55	21 (64)
	≥55	12 (36)
Selective Neck Dissection		
	with TT	24 (73)
	without TT	9 (27)
Mean operating time (range), min		226 (120–330)
Pathology		
	Classic papillary	26 (79)
	Follicular variant	7 (21)
	Papillary thyroid microcarcinoma	4 (12)
	Unifocal PTC	21 (64)
	Multifocal PTC	8 (24)
	T1a	9 (27)
	T1b	5 (15)
	T2	3 (9)
	T3	16 (49)
	N1b	33 (100)
Mean number of nodes retrieved in SND (range) *		10.4 (4–24)
	IIa	3.9 (2–7)
	IIb	4.3 (2–6)
	III	4 (2–7)
	IV	5 (2–10)
	V	2.2 (1–3)
Mean number of nodes positive at pathology (range) *		2.61 (1–5)
Mean Follow-up (range), years		59 (17–100)
Recurrence		2 (6)

* Does not include nodes retrieved from central compartment dissection.

**Table 2:** Nodal compartments detected as pathologic, respectively, at the preoperative ultrasound scan (PreUS), intraoperative ultrasound scan (IOUS) and pathological examination of the resected specimen, along with the metrics of the diagnostic power of PreUS and IOUS. LN: lymph nodes, PPV: positive predictive value, NPV: negative predictive value, FP: false positive and FN: false negative. In all the cases, the compartments suspect for metastasis at IOUS matched the compartments detected as metastatic at PreUS before additional compartments, potentially metastatic, were indicated by IOUS.

		Positive Compartment Nodes Detected, by Modality, *n*. pts (%)	*p* * (PreUS vs. IOUS)	Additional Compartments Detected at IOUS Confirmed at Pathology N. pts (%)	Mean Number of LN Retrieved in Additional Compartments Detected by IOUS	Sensitivity % (95% CI)	Specificity	Accuracy	PPV	NPV	FP Rate	FN Rate
	All cases	33		8 (24.2)	4.3							
N level												
IIA	PreUS	16 (48)	*p* < 0.0001			94.1 (0.69–1)	100 (0.77–1)	96.9	100 (0.76–1)	94.1 (0.70–1)	0 (0–0.24)	5.9 (0.71–1)
	IOUS	17 (52)		1/33 (3)	4	100 (0.77–1)	100 (0.76–1)	100	100 (0.77–1)	100 (0.76–1)	0 (0–0.23)	0 (0–0.24)
	Pathol	17 (52)										
IIB	PreUS	3 (9)	*p* = 0.006			42.9 (0.11–0.8)	100 (0.84–1)	87.8	100 (0.31–1)	86.7 (0.68–0.96)	0 (0–0.69)	13.3 (0.04–0.31)
	IOUS	7 (21)		4/33 (12.1)	5.3	100 (0.56–1)	100 (0.84–1)	100	100 (0.56–1)	100 (0.84–1)	0 (0–0.43)	0 (0–0.16)
	Pathol	7 (21)										
III	PreUS	27 (82)	*p* < 0.0001			100 (0.84–1)	85.7 (0.42–1)	96.9	96.3 (0.79–1)	100 (0.52–1)	0.03 (0.01–0.21)	0 (0–0.48)
	IOUS	27 (82)		0/33 (0)		100 (0.84–1)	85.7 (0.42–1)	96.9	96.3 (0.79–1)	100 (0.52–1)	0.03 (0.01–0.21)	0 (0–0.48)
	Pathol	26 (79)										
IV	PreUS	24 (72)	*p* < 0.0001			96 (0.78–1)	100 (0.6–1)	96.9	100 (0.83–1)	89 (0.51–1)	0 (0–0.17)	0.11 (0.01–0.5)
	IOUS	25 (76)		1/33 (3)	7	100 (0.83–1)	89 (0.6–1)	100	100 (0.83–1)	100 (0.6–1)	0 (0–0.16)	0 (0–0.4)
	Pathol	25 (76)										
V	PreUS	5 (15)	*p* < 0.0001			71.4 (0.3–0.95)	100 (0.84–1)	93.9	100 (0.46–1)	92.9 (0.75–0.99)	0 (0–0.54)	0.07 (0.01–0.25)
	IOUS	8 (24)		2/33 (6)	2.5	100 (0.56–1)	96.1 (0.78–1)	100	87.5 (0.47–1)	100 (0.83–1)	0.125 (0.01–0.53)	0 (0–0.17)
	Pathol	7 (21)										

* chi-square test of independenc

## Data Availability

The data presented in this study are available on request from the corresponding author. The data are not publicly available due to privacy and ethical restrictions.
